# Early Disturbance of Lymphatic Transport as a Risk Factor for the Development of Breast-Cancer-Related Lymphedema

**DOI:** 10.3390/cancers15061774

**Published:** 2023-03-15

**Authors:** Sarah Thomis, Nele Devoogdt, Beate Bechter-Hugl, Inge Fourneau

**Affiliations:** 1 Centre for Lymphedema, Department of Vascular Surgery, UZ Leuven—University Hospitals Leuven, 3000 Leuven, Belgium; 2 Research Unit Vascular Surgery, Department of Cardiovascular Sciences, KU Leuven—University of Leuven, 3000 Leuven, Belgium; 3 Department of Rehabilitation Sciences, KU Leuven—University of Leuven, 3000 Leuven, Belgium

**Keywords:** lymphedema, risk, ICG lymphofluoroscopy, near-infrared fluorescence, early detection

## Abstract

**Simple Summary:**

Lymphedema is a chronic debilitating condition that requires continuous attention. ICG fluoroscopy shows a detailed superficial lymphatic architecture, can provide additional information regarding the functionality of lymphatic transport and can show early abnormalities of the lymphatic system. The aim of this study is to investigate whether his early disturbance of lymphatic transport visualized by lymphofluoroscopy is a risk factor for the development of breast-cancer-related lymphedema (BCRL). No such research has been conducted to date. All patients scheduled for breast cancer surgery with unilateral axillary lymph node dissection or sentinel node biopsy were considered. Patients were assessed at baseline and 1, 3, 6, 9, 12, 18, 24 and 36 months postoperatively. During each visit, a clinical assessment was performed to determine the presence of clinical lymphedema (≥5% increase in relative arm volume difference compared to the baseline value). Variables related to (1) the disturbance of lymphatic transport, (2) the demographics and general health of the patient and (3) the breast cancer and treatment of the patient were also investigated. Early disturbance of lymphatic transport visualized by lymphofluoroscopy was identified as a risk factor for the development of clinical BCRL. Age and axillary lymph node dissection were withheld as independent risk factors. A surveillance program of these high-risk patients with lymphofluoroscopy can be useful to identify lymphedema in a subclinical stage and prevent development to more advanced stages.

**Abstract:**

Introduction: Breast-cancer-related lymphedema (BCRL) is a frequently occurring and debilitating condition. When lymphedema is diagnosed late, treatment can be expected to be less effective. Lymphofluoroscopy can provide details about the superficial lymphatic architecture and can detect an early disturbance of lymphatic transport (i.e., dermal backflow) before the lymphedema is clinically visible. The main objective of this study is to investigate whether this early disturbance of lymphatic transport visualized by lymphofluoroscopy is a risk factor for the development of BCRL. Methodology: All patients scheduled for unilateral breast cancer surgery with axillary lymph node dissection or sentinel node biopsy were considered. Patients were assessed at baseline and 1, 3, 6, 9, 12, 18, 24 and 36 months postoperatively. During each visit, a clinical assessment was performed to determine the volume difference between both arms and hands (through circumference measurements and water displacement). Clinical BCRL was defined as a ≥5% increase in relative arm volume difference compared to the baseline value. Variables related to (1) the disturbance of lymphatic transport (through lymphofluoroscopy), (2) the demographics and general health of the patient and (3) the breast cancer and treatment of the patient were collected. Results: We included data of 118 patients in the present study. Thirty-eight patients (39.8%) developed BCRL. Early disturbance of lymphatic transport was identified as a risk factor for the development of clinical BCRL (HR 2.808). Breast-cancer- and treatment-related variables such as axillary lymph node dissection (ALND) (HR 15.127), tumor stage (HR 1.745), mastectomy (HR 0.186), number of positive lymph nodes (HR 1.121), number of removed lymph nodes (HR 1.055), radiotherapy of the axilla (HR 2.715), adjuvant taxanes (HR 3.220) and postsurgical complications (HR 2.590) were identified as significant risk factors for the development of BCRL. In the multivariate analysis, age and ALND were withheld as independent risk factors for the development of BCRL. Conclusion: Lymphofluoroscopy can identify an early disturbance of lymphatic transport after breast cancer treatment. Patients with an early disturbance of lymphatic transport are considered to be a high-risk group for the development of BCRL. This study also confirms that age and ALND are predictors for the development of BCRL. Therefore, a surveillance program of these patients with lymphofluoroscopy could be useful to identify lymphedema in subclinical stages.

## 1. Introduction

Despite the availability of less invasive surgical techniques and treatment options, breast-cancer-related lymphedema (BCRL) remains one of the most important and feared complications after breast cancer treatment [1]. Breast cancer survivors have a lifelong risk of developing lymphedema, with the incidence rate ranging from 5.6% to 63.4% [2]. Surgical and non-surgical techniques have been implemented for the prevention of the development of lymphedema [3,4,5], but further research is needed.

A clinical stage system can be used based on the history and clinical examination of the patient [1]. Four stages are defined: stage 0 refers to a subclinical state, where edema is not yet visible despite impaired lymphatic transport; stage I refers to an early accumulation of fluid; stage IIa represents swelling that does not subside with limb elevation manifest pitting; stage IIb is characterized by the disappearance of pitting and fibrosis, together with the emergence of fat; and stage III is the most advanced form, with skin abnormalities and further fat accumulation and fibrosis of the tissue.

Clinical assessment tools such as tissue dielectric constant, bioelectrical impedance spectroscopy, circumference measurement, perometric measurements and the water displacement method can be used to detect lymphedema [6,7]. Preoperative and postoperative measurement at regular times are needed to detect lymphedema early, but there still is no consensus on the threshold defining subclinical lymphedema [6]. In some studies, a threshold of ≥3% volume increase compared to preoperative values is defined as subclinical BCRL [8]. A threshold of ≥5% volume increase is used to define clinical BCRL. Other measurement techniques can assess fluid in the tissue, either in the extracellular space (bioelectrical impedance spectroscopy) [9] or the skin (tissue dielectric constant) [10].

Lymphofluoroscopy, or near-infrared fluorescence lymphatic imaging, is an imaging technique that visualizes the disturbance of superficial lymphatic transport. Three patterns of dermal backflow (splash, stardust and diffuse) are described according to the severity of the disturbance [11]. According to Akita et al., this imaging technique can be used for early detection of BCRL (=subclinical BCRL) [12]. They included 196 patients planned for surgical treatment of breast cancer, of which 25% developed a dermal backflow pattern within the first year after the surgery.

A number of risk factors for the development of clinical BCRL have been investigated. These can be categorized as risk factors related to demographics and general health (such as body mass index (BMI), age, race or diabetes) and risk factors related to the treatment (such as type of surgery, type of lymph node dissection, chemotherapy, radiotherapy, tumor stage and number of positive lymph nodes). A higher age and a higher BMI have been proven to be associated with a higher risk for the development of clinical BCRL. In some studies, a low level of physical activity [13], hypertension [14], black race [14] and a low level of education [15] were associated with a higher risk of BCRL. Modified radical mastectomy (versus breast-conserving surgery), axillary lymph node dissection (ALND) versus sentinel lymph node dissection (SLND), radiotherapy, chemotherapy and postsurgical complications [16] have been described as treatment-related risk factors for the development of clinical BCRL [2,17,18,19]. Higher tumor stage and higher number of positive lymph nodes are known risk factors related to breast cancer [2,19].

Whether early disturbance of lymphatic transport visualized by lymphofluoroscopy is a risk factor for the development of clinical BCRL has not been investigated to date.

The primary aim of this study is to investigate whether early disturbance of lymphatic transport visualized by lymphofluoroscopy is a risk factor for the development of clinical BCRL. The secondary aim is to investigate whether demographic, general health and treatment-related variables found in the literature can be confirmed in the present study.

## 2. Methodology

### 2.1. Trial Design

The present study is a prospective cohort study that is part of the Dearly trial (Determining the role of pre-existing factors, early diagnostic options and early treatment in the development of BCRL) [20]. The study was approved by the Ethical Committee of the University Hospitals Leuven (S-number 60382, EudraCT number 2017-002306-12). The study has been registered at clinicaltrials.gov (NCT 03210311).

### 2.2. Participants

The recruitment of subjects started in November 2017 and ended in May 2019. All consecutive breast cancer patients who were scheduled for surgery for primary breast cancer were asked to participate. All patients were recruited in the Multidisciplinary Breast Centre of the University Hospitals Leuven, Belgium. Inclusion criteria were (1) age ≥18 y, (2) women/men with primary unilateral breast cancer and scheduled for axillary lymph node dissection (ALND) or sentinel lymph node biopsy (SLNB), (3) oral and written approval of informed consent and (4) understanding Dutch. Exclusion criteria were (1) edema of the upper limb from other causes, (2) not being able to participate during the entire study period, (3) mentally or physically unable to participate in the study, (4) contraindication for the use of indocyanine green (ICG) or (5) metastatic disease.

All patients received oral and written information. All included patients signed an informed consent form prior to the start of the study.

### 2.3. Data Collection

All assessments were performed at baseline and 1, 3, 6, 9, 12, 18, 24 and 36 months postoperatively.

### 2.4. Development of Clinical BCRL

During the various follow-up visits, the presence of clinical BCRL was evaluated. Circumference measurements at the olecranon and 4, 8, 12, 16 and 20 cm above and under the olecranon were performed in the affected and healthy arms [21]. The volume of the arm segments was calculated using the formula of the truncated cone (V = 4 × (C_1_^2^ + C_1_C_2_ + C_2_^2^)/12π, where V is the volume, C_1_ is the upper circumference and C_2_ is the lower circumference of each segment) [22]. The volume of the arm (without the hand) is the sum of the volume of the different arm segments. The hand volume of both sides was determined by the water displacement method using the most distal skinfold at the wrist as the reference point [23]. The volume of the arm was determined as the sum of the volume of the arm (without hand) and the volume of the hand.

Clinical BCRL was defined as a ≥5% increase in relative arm volume difference compared to the baseline value. The relative arm volume difference was calculated as the absolute arm volume difference between the affected side and the healthy side divided by the absolute arm volume of the health side multiplied by 100, and the absolute arm volume was calculated as the sum of the volumes of the different arm segments and the hand volume. Clinical BCRL was scored positive when observed at 36 months or earlier.

### 2.5. Lymphatic-Transport-Related Variable

All lymphofluoroscopic assessments were performed by one person (ST) who was blinded to the participants’ data.

During lymphofluoroscopy, ICG was injected intradermally in the first and fourth webspace of the hand on the affected side. The same procedure described in a previous publication [20] was used for lymphofluoroscopy.

The presence of dermal backflow was scored 0 if a normal (linear) pattern was seen and 1 if an abnormal pattern (splash, stardust or diffuse) at least the size of a EUR 2 coin was seen. Early disturbance of lymphatic transport was defined as present if there was at least one occurrence of lymphofluoroscopy abnormality before the first occurrence of clinical BCRL. Patients were divided into four different groups: patients who developed clinical BCRL with and without early disturbance and patients who developed no clinical BCRL with or without early disturbance of lymphatic transport.

### 2.6. Demographic and General Health-Related Variables

Demographic variables (age, dominance and BMI) and general health-related variables (diabetes, hypertension, hypothyroidism, hyperthyroidism, chronic heart failure, chronic renal failure and history of infection or trauma in the affected limb) were collected by interview with the patients.

The physical activity level was assessed at baseline using the International Physical Activity Questionnaire (IPAQ long version). This questionnaire comprises a set of 5 activity domains asked independently [24]. According to the scoring, three levels of physical activity were assigned: low (<600 metabolic equivalent (MET) min/week), moderate (<3000 MET min/week) and high (>3000 MET min/week).

### 2.7. Breast-Cancer- and Treatment-Related Variables

Data related to the breast cancer such as tumor stage, type of cancer and the number of excised and positive lymph nodes were recorded according to the pathology report in the patient’s electronic medical file.

Treatment-related variables consisting of the type of surgery, chemotherapy, radiotherapy, hormone therapy and postsurgical complications were identified by notes in the electronic medical file of the patient.

### 2.8. Statistical Methods

Group comparisons were performed using Fisher’s exact test for nominal variables or a Mann–Whitney U test for continuous or ordinal variables. Most of the variables were nominal variables. The variables age, BMI, physical activity level, number of removed lymph nodes and number of positive lymph nodes were analyzed as continuous variables. Tumor stage was analyzed as an ordinal variable.

Logistic regression analyses (=univariate analyses) were applied to investigate the prognostic effect of possible risk factors on the development of clinical BCRL (yes/no). The results were reported as hazard ratios (HRs) with 95% confidence intervals.

Thereafter, a forward stepwise model selection procedure was applied to develop a multivariable model of independent risk factors.

All reported *p*-values are two-sided. Analyses were performed using SAS software (version 9.4 of the SAS System for Windows).

## 3. Results

### 3.1. Description of Participants

A total of 118 patients were enrolled for this trial. The mean age of the patients was 55.6 (SD 12.0), and the mean BMI was 25.9 (SD 4.9). ALND was performed in 70 patients (59%), and 48 patients (41 %) underwent SLNB. A total of 83 patients (70%) underwent a mastectomy, and 35 patients (30%) underwent a breast-conserving surgery. Detailed patient characteristics are summarized in Table 1.

Of the 118 patients in the study, 47 (39.83%) developed clinical BCRL after a follow-up time of 36 months. A total of 10 patients developed BCRL after 1 month, 7 patients developed BCRL after 3 months, 9 patients developed BCRL after 6 months, 7 patients developed BCRL after 9 months, 7 patients developed BCRL after 12 months, 3 patients developed BCRL after 24 months and 4 patients developed BCRL after 36 months. The median absolute volume difference of the arm in the group of patients with clinical BCRL was comparable between with that of patients who did not develop early disturbance of lymphatic transport and the patients who did develop early disturbance (175 mL versus 152 mL, respectively, *p* = 1.0000) (Table 2).

### 3.2. Lymphatic-Transport-Related Variable

**Figure 1:**
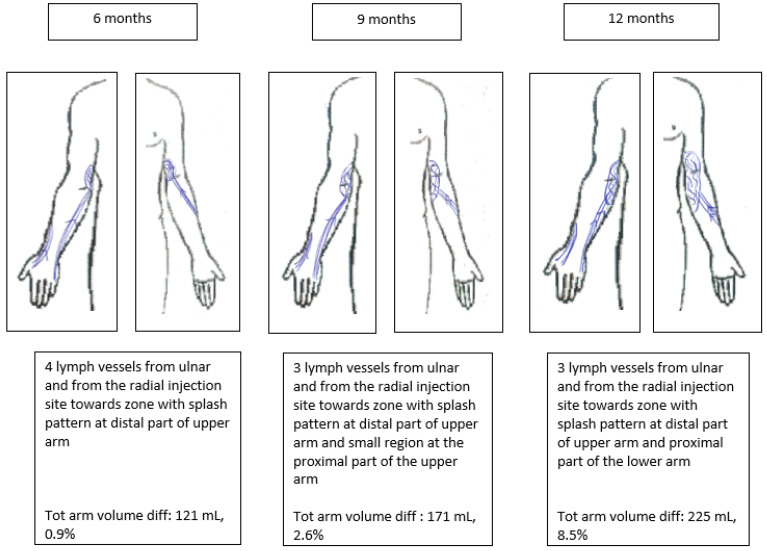
Lymphofluoroscopy of a patient with early disturbance 6, 9 and 12 months postoperatively and with development of lymphedema 12 months postoperatively.

For this variable, only data of 112 patients could be included. Six patients missed a lymphofluoroscopic evaluation during follow-up visits, mainly due to COVID-19. Preoperative lymphofluoroscopy was normal in all patients. In forty-one patients, an early disturbance of lymphatic transport at any time point was visualized. Of these 41 patients, 17 patients developed clinical BCRL during the follow-up after 36 months. Additionally, 13 patients developed early disturbance after 1 month, 15 after 3 months, 5 after 6 months, 6 after 9 months, 1 after 12 months and 1 after 24 months. Twenty-four patients with early disturbances did not develop clinical BCRL during the follow-up after 36 months. Figure 1 shows an example of a patient who showed early disturbance visualized by lymphofluoroscopy after 6 and 9 months; however, at that time point, no clinical BCRL was present. After 12 months, this patient developed clinical BCRL with a relative volume difference of 8.5%. In the univariate analyses, the presence of early disturbance of lymphatic transport was a significant predictor for the development of clinical BCRL (*p* = 0.0052) (Table 3).

### 3.3. Demographic and General Health-Related Variables

In this analysis, age and BMI did not significantly differ between the groups with and without clinical lymphedema (*p* = 0.1838 and *p* = 0.3509, respectively; Table 3).

Univariate analysis showed that general health-related variables such as diabetes, hypertension, hypothyroidism, hyperthyroidism, chronic heart failure, chronic renal failure and previous injury/infection did not significantly differ between the two groups (Table 3). Dominance was not found to be a risk factor for the development of clinical lymphedema in this study. The physical activity score before surgery, which was assessed in 113 patients (data from 5 patients were missing), did not significantly differ between the two groups (*p* = 0.2385).

### 3.4. Breast-Cancer- and Treatment-Related Variables

Regarding the data from the pathology reports (Table 3), the type of cancer was not a risk factor for the development of clinical BCRL. Patients with a higher tumor stage were more prone to develop clinical BCRL (*p* = 0.0001) than patients with a lower tumor stage, and patients who underwent a mastectomy (70%) had a higher risk of development of BCRL than patients who underwent a breast-conserving surgery (30%) (*p* = 0.0013). The number of removed lymph nodes, as well as the number of positive lymph nodes, was differed significantly in the group with clinical BCRL compared to the group without (both *p* < 0.0001). The extent of lymph node dissection (ALND versus SLNB) also significantly differed between the two groups (*p* < 0.0001). Postsurgical complications such as infection were also more present in the group with clinical BCRL (compared to the group without BCRL) (*p* = 0.0077). Lymphedema was more likely to occur in patients who received adjuvant taxanes (*p* = 0.0005) and radiotherapy of the axilla (*p* = 0.0228). Hormone therapy (tamoxifen and aromatase inhibitors) did not differ significantly between the two groups.

### 3.5. Multivariate Analysis

After multivariate analysis, the following variables were positively associated with BCRL: higher age and receiving ALND (vs SNB) (Table 4). The remaining variables did not have additional prognostic value.

## 4. Discussion

This is the first trial investigating the role of early disturbance of lymphatic transport in the development of BCRL. 

### 4.1. Lymphatic-Transport-Related Variable Associated with BCRL

In this study, early disturbance was visible in 41 of the 112 patients (36.61%). Our data showed that early disturbance visualized by lymphofluoroscopy is a risk factor for the development of BCRL. However, early disturbance visualized by lymphofluoroscopy was not withheld as an independent risk factor in the multivariate analysis. 

Twenty-four patients with early disturbance did not develop clinical BCRL within the 36 months of follow-up. It is possible that some of these patients will develop clinical BCRL in a later time. Another possible reason is that this early disturbance is a way to reroute the lymphatic flow, not necessarily resulting in clinical BCRL but merely in subclinical BCRL. Subclinical lymphedema is difficult to assess with other clinical measurements tool according to our previous study [25] and the study by Jørgensen [26]. Some patients had disturbances visualized by lymphofluoroscopy, but no volume differences were present.

Akita et al. investigated the presence of dermal backflow in 189 patients who underwent breast cancer surgery (ALND and SLNB). A total of 50 of 196 arms (25.5%) showed an abnormal pattern within the first year after breast cancer treatment, and no significant change in volume was seen before the presence of disturbance visualized by lymphofluoroscopy [27].

In a recent study, Liu performed a retrospective analysis of 179 patients. In this study, lymphatic disorder visualized by ICG lymphofluoroscopy was withheld as a risk factor for BCRL [28]. However, this risk factor was not analyzed in the multivariate analysis, and no baseline data were used. Another study that included only patients who received ALND and regional nodal radiotherapy concluded that BCRL can be predicted by ICG lymphofluoroscopic findings, enabling an early start of treatment [29]. The follow-up of this study was 18 months, and no univariate analysis was performed; however, accuracy, sensitivity and specificity of lymphofluoroscopy for BCRL were calculated.

To date, other imaging techniques such as lymphoscintigraphy have not been able to detect early disturbance [30,31].

### 4.2. Demographic and General Health-Related Variables Associated with Clinical BCRL

Demographics such as BMI showed no significance in our study, as confirmed in several other studies [32,33]. Our data did show that there was an HR > 1, so a higher BMI is associated with a higher risk for the development of BCRL. In a systematic review by DiSipio [2], a high BMI was identified as a risk factor with a high level of evidence. One of the difficulties in analyzing the data is that in different studies, different variables are used. BMI is sometimes used as a continuous variable and sometimes grouped in different categories. Other variables such as age, education and employment status were weak or inconclusive according to a review by DiSipio [2]. In our study, age was not a risk factor in the univariate analysis, although in the multivariate analysis, age was withheld as an independent risk factor, so its effect was concealed in the univariate analysis.

General health-related variables such as diabetes, thyroidism, hypertension and previous injury/infection have been investigated in different studies but were not identified as significant risk factors [34,35,36,37].

Recent studies have demonstrated that by activating the muscle pump through exercise, lymphatic transport is promoted. [38] Baumann et al. [39] also reported that exercise might also have a preventive effect on the development of lymphedema. In this study. we could not find an association between a low preoperative physical activity level and the development of lymphedema. This finding is similar to those reported by other authors [40,41]. According to the review by DiSipio, there is a moderate level of evidence that not participating in regular physical activity is a possible risk factor for the development of BCRL [2].

### 4.3. Breast-Cancer- and Treatment-Related Variables Associated with Clinical BCRL

More invasive surgical procedures are more prone to result in the development of clinical BCRL than less invasive procedures such as breast-conserving treatment. The reason for this is not totally clear, but the fact that more advanced cancer requires more invasive treatment such as a mastectomy combined with an ALND may explain this phenomenon, as confirmed in other studies [19]. ALND and a higher number of removed lymph nodes were identified as important risk factors in this study. The severity of the cancer can also be expressed as a higher tumor stage, which was also confirmed as a risk factor for the development of lymphedema. Therefore, patients with more advanced breast cancer have the highest risk of developing clinical lymphedema. Three out of the forty-seven patients (6.38%) who underwent sentinel lymph node biopsy developed clinical lymphedema, in accordance with the review by DiSipio, who reported 5.6% [2].

Postsurgical complications such as seroma were significantly different between the two groups in the current study. Other studies have identified the duration of the seroma as an important risk factor [42,43,44]. However, postsurgical infection is most often reported as a risk factor for the development of BCRL [15,45,46]. Prevention of postoperative seromas and infections remains an important issue, and further research is needed [47].

Radiotherapy of the axilla was seen as a risk factor. Other studies examining the effect of radiotherapy on the development of lymphedema, specifically radiotherapy of the axilla, confirm this [48,49]. Radiotherapy not only blocks the lymph vessels but also compresses them by radiation fibrosis. Radiation of the axilla is indicated if a patient has ≥ 4 positive lymph nodes with capsule breakthrough, if the tumor is left in the axilla and if not enough lymph nodes are removed during ALND (<6 LN). Therefore, the number of positive lymph nodes is also related to the development of BCRL, again confirming that patients with more advanced cancers are at the greatest risk. Another indication for radiotherapy of the axilla is one or two positive lymph nodes after SLNB. The AMAROS trial found significant less lymphedema in the radiotherapy and SLNB group versus the ALND group [50].

Chemotherapy is mentioned as a risk factor in several studies, specifically taxanes [51,52,53]. In a study by Cariati [52], women receiving taxanes in the adjuvant setting were nearly twice as likely to develop BCRL as patients receiving non-taxane-based adjuvant chemotherapy, although this did not reach statistical significance.

Hormone therapy was not identified as a risk factor for the development of BCRL, in line with different studies investigating this phenomenon [43,54,55].

### 4.4. Strenghts and Limitations

A strength of this study is the prospective design, as preoperative and postoperative data of 118 patients were included in this trial. Another strength is the follow-up time of 36 months. A clear and up-to-date definition of BCRL and early disturbance was used.

A limitation of this study is that the physical activity score was assessed at baseline and not at the follow-up points. The physical activity score could have been diminished at a later time point, especially in patients who still needed chemotherapy and/or radiotherapy, or could have been increased if patients participated in an exercise program to reduce chemo symptoms. Other variables such as BMI may have also changed during the postoperative phase.

### 4.5. Clinical Implications

A previous study [25] analyzed the agreement between early disturbance visualized by lymphofluoroscopy and other widely used clinical assessment tools. None of the clinical assessment tools could predict early disturbance visualized by lymphofluoroscopy. This study shows that early disturbance visualized by lymphofluoroscopy is a risk factor for the development of BCRL. Screening high-risk patients (e.g., ALND and higher age) with lymphofluoroscopy will make it possible to start treatment before clinical lymphedema is present.

## 5. Conclusions

In this prospective study, 39.8% of the 118 patients developed clinical BCRL after a follow-up of 36 months. Early disturbance visualized by lymphofluoroscopy is a risk factor for the development of BCRL. However, the major predictive factors in the whole group of breast cancer patients (who received treatment with ALND or SNB) are ALND and a higher age. Surveillance of this group of patients with lymphofluoroscopy on a regular basis during the postoperative follow-up period could prevent clinical BCRL.

## Figures and Tables

**Table 1:** Variables of the study participants.

Variable	Without Clinical BCRL after 3 Years	With Clinical BCRL after 3 Years	*p*-Value
**Demographic and general health-related variables**			
	N = 71	N = 47	
**Age**	54.6 (±11.8) *	56.9 (±12.7) *	0.394
**BMI**	25.7 (±4.9) *	26.2 (±4.9) *	0.451
**Surgery on dominant side**			
No	34 (47.89%)	24 (51.06%)	0.851
Yes	37 (52.11%)	23 (48.94%)	
**Diabetes**			
No	68 (95.77%)	45 (95.74%)	1.000
Yes	3 (4.23%)	2 (4.26%)	
**Hypothyroidism**			
No	68 (95.77%)	40 (85.11%)	0.087
Yes	3 (4.23%)	7 (14.89%)	
**Hyperthyroidism**			
No	68 (95.77%)	46 (97.87%)	1.000
Yes	3 (4.23%)	1 (2.13%)	
**Arterial hypertension**			
No	57 (80.28%)	36 (76.60%)	0.651
Yes	14 (19.72%)	11 (23.40%)	
**Chronic heart failure**			
No	70 (98.59%)	46 (97.87%)	1.000
Yes	1 (1.41%)	1 (2.13%)	
**Chronic renal failure**			
No	71 (100%)	46 (97.87%)	0.398
Yes	0 (0%)	1 (2.13%)	
**Previous injury/infection**			
No	65 (91.55%)	43 (91.49%)	1.000
Yes	6 (8.45%)	4 (8.54%)	
	N = 69	N = 44	
**Physical activity score**			
Low	7 (10.14%)	6 (13.64%)	0.630
Moderate	29 (42.03%)	21 (47.73%)	
High	33 (47.83%)	17 (38.64%)	
**Breast-cancer- and treatment-related variables**			
	N = 71	N = 47	
**Type of cancer**			
Ductal	58 (81.69%)	37 (78.72%)	0.586
Lobular	8 (11.27%)	8 (17.02%)	
Other	5 (7.04%)	2 (4.26%)	
**Tumor stage**			
Tis	2 (2.82%)	0 (0%)	0.002
T1	30 (42.25%)	10 (21.28%)	
T2	31 (43.66%)	18 (38.30%)	
T3	6 (8.45%)	11 (23.40%)	
T4	2 (2.82%)	8 (17.02%)	
**Type of surgery**			
Mastectomy	40 (56.34%)	43 (91.49.84%)	<0.001
Breast-conserving surgery	31 (43.66%)	4 (8.51%)	
**Extent of LN dissection**			
SLNB	45 (63.38%)	3 (6.38%)	<0.001
ALND	26 (36.62%)	44 (93.62%)	
**Number of removed LNs**	8.68 (±10.351) *	18.45 (±8.235) *	<0.001
**Number of positive LNs**	0.85 (±2.494) *	3.26 (±3.26) *	<0.001
**Postsurgical complications**			
No	35 (49.30%)	10 (21.28%)	0.003
Yes	36 (50.70%)	37 (78.72%)	
**RT axilla**			
No	69 (97.18%)	41 (87.23%)	0.058
Yes	2 (2.82%)	6 (12.77%)	
**Taxanes**			
No	45 (63.38%)	12 (25.53%)	<0.001
Yes	26 (36.62%)	35 (74.47%)	
**Tamoxifen**			
No	56 (78.87%)	38 (80.85%)	1.000
Yes	15 (21.13%)	9 (19.15%)	
**AI**			
No	25 (35.21%)	12 (25.53%)	0.314
Yes	46 (64.79%)	35 (74.47%)	

* Mean (SD). BMI: body mass index; LNs: lymph nodes; RT: radiotherapy; AI: aromatase inhibitor; ALND: axillary lymph node dissection; SLNB: sentinel lymph node biopsy.

**Table 2:** Arm volume of affected side and absolute and relative arm volume differences for the different groups.

	Patients with Clinical BCRL N = 42			Patients without Clinical BCRL N = 70		
	Without Early Disturbance	With Early Disturbance	*p*-Value	Without Early Disturbance	With Early Disturbance	*p*-Value
	N = 25	N = 17		N = 46	N = 24	
Total volume Median (range)	2632 mL (1477;4548)	2537 mL (1821;3892)	0.898	2554 mL (1641;3952)	2490 mL (1709;3695)	0.590
Absolute volume difference sum Median (range)	175 mL (−52.87;420)	152 mL (19.94;500)	1.000	−28 mL (−250;163)	−43 mL (−231;197)	0.758
Relative volume difference percentage Median (range)	7.61% (−1.77;18.78)	6.34% (0.90;23.38)	0.778	−0.86% (−10,91;6.62)	−1.69% (−9.11;8.56)	0.806

**Table 3:** Univariate analysis of variables.

Variable	HR (95% CI)	*p*-Value	Number of Patients
Early disturbance lymphofluoroscopy versus no early disturbance lymphofluoroscopy	2.808 (1.362;5.791)	**0.0052**	112
**Demographic and general health-related variables**			
Age, continuous	1.017 (0.992;1.042)	0.1838	118
BMI, continuous	1.028 (0.970;1.089)	0.3509	118
Treatment on dominant side versus non-dominant side	0.895 (0.505;1.586)	0.7033	118
Hypertension versus no hypertension	1.219 (0.620;2.395)	0.5658	118
Diabetes versus no diabetes	1.327 (0.321;5.490)	0.6964	118
Hypothyroidism versus no hypothyroidism	2.070 (0.926;4.626)	0.0762	118
Hyperthyroidism versus no hyperthyroidism	0.503 (0.069;3.649)	0.4967	118
Chronic heart failure versus no chronic heart failure	1.277 (0.176;9.282)	0.8091	118
Chronic renal failure versus no chronic renal failure	2.432 (0.334;17.703)	0.3801	118
Previous injury/infection versus no previous injury/infection	1.113 (0.399;3.103)	0.8374	118
Physical activity score, continuous	0.768 (0.496;1.191)	0.2385	113
**Breast-cancer- and treatment-related variables**			
Type of cancer		0.4767	118
Tumor stage	1.745 (1.317;2.313)	**0.0001**	118
BCS versus ME	0.186 (0.067;0.519)	**0.0013**	118
Number of removed LNs, continuous	1.055 (1.030;1.080)	**<0.0001**	118
Number positive LNs, continuous	1.121 (1.060;1.186)	**<0.0001**	118
Postsurgical complications versus no postsurgical complications	2.590 (1.286;5.213)	**0.0077**	118
RT axilla versus no RT axilla	2.715 (1.149;6.416)	**0.0228**	118
Taxanes versus no taxanes	3.220(1.669;6.210)	**0.0005**	118
Tamoxifen versus no tamoxifen	0.874 (0.422;1.807)	0.7160	118
AI versus no AI	1.400 (0.726;2.697)	0.3152	118
ALND versus SLNB	15.127 (4.684;48.830)	**<0.0001**	118

HR: hazard ratio; CI: confidence interval; ME: mastectomy; BCS: breast-conserving surgery; LNs: lymph nodes; RT: radiotherapy; AI: aromatase inhibitor; ALND: axillary lymph node dissection; SLNB: sentinel lymph node biopsy; HR >(<) 1: higher (lower) risk with increasing predictor level/for first-category; categorical variables with >2 levels: the global *p*-value is given.

**Table 4:** Multivariate analysis of variables.

Variable	Test	HR (95% CI)	*p*-Value	Number of Patients
ALND	ALND vs. SLNB	19.958 (6.108;65.217)	<0.0001	118
Age	+1 year	1.038 (1.015;1.062)	0.0011	118

ALND: axillary lymph node dissection; SLNB: sentinel lymph node biopsy; HR: hazard ratio; CI: confidence interval; HR >(<) 1: higher (lower) risk with increasing predictor level/for the first category.

## Data Availability

The data presented in this study are available upon request from the corresponding author. The data are not publicly available due to privacy restrictions.

## References

[1] (2020). The diagnosis and treatment of peripheral lymphedema: 2020 Consensus Document of the International Society of Lymphology. Lymphology.

[2] DiSipio T., Rye S., Newman B., Hayes S. (2013). Incidence of unilateral arm lymphoedema after breast cancer: A systematic review and meta-analysis. Lancet Oncol..

[3] Markkula S.P., Leung N., Allen V.B., Furniss D. (2019). Surgical interventions for the prevention or treatment of lymphoedema after breast cancer treatment. Cochrane Database Syst. Rev..

[4] Ciudad P., Escandón J.M., Bustos V.P., Manrique O.J., Kaciulyte J. (2022). Primary Prevention of Cancer-Related Lymphedema Using Preventive Lymphatic Surgery: Systematic Review and Meta-analysis. Indian J. Plast. Surg..

[5] Devoogdt N., Christiaens M.R., Geraerts I., Truijen S., Smeets A., Leunen K., Neven P., Van Kampen M. (2010). Abstract S5-3: Is Manual Lymph Drainage Applied after Axillary Lymph Node Dissection for Breast Cancer Effective To Prevent Arm Lymphoedema? A Randomised Controlled Trial. Cancer Res..

[6] Shah C., Arthur D.W., Wazer D., Khan A., Ridner S., Vicini F. (2016). The impact of early detection and intervention of breast cancer-related lymphedema: A systematic review. Cancer Med..

[7] Gencay Can A., Eksioglu E., Cakci F.A. (2019). Early Detection and Treatment of Subclinical Lymphedema in Patients with Breast Cancer. Lymphat. Res. Biol..

[8] Stout N.L., Pfalzer L., Levy E., McGarvey C., Gerber L., Springer B., Soballe P. (2011). Five year preliminary outcomes of a prospective surveillance model to reduce upper extremity morbidity related to breast cancer treatment. Cancer Res..

[9] Soran A., Ozmen T., McGuire K.P., Diego E.J., McAuliffe P.F., Bonaventura M., Ahrendt G.M., Degore L., Johnson R. (2014). The importance of detection of subclinical lymphedema for the prevention of breast cancer-related clinical lymphedema after axillary lymph node dissection; A prospective observational study. Lymphat. Res. Biol..

[10] De Vrieze T., Gebruers N., Nevelsteen I., De Groef A., Tjalma W.A.A., Thomis S., Dams L., Van der Gucht E., Penen F., Devoogdt N. (2020). Reliability of the MoistureMeterD Compact Device and the Pitting Test to Evaluate Local Tissue Water in Subjects with Breast Cancer-Related Lymphedema. Lymphat. Res. Biol..

[11] Yamamoto T., Yamamoto N., Doi K., Oshima A., Yoshimatsu H., Todokoro T., Ogata F., Mihara M., Narushima M., Iida T. (2011). Indocyanine green-enhanced lymphography for upper extremity lymphedema: A novel severity staging system using dermal backflow patterns. Plast. Reconstr. Surg..

[12] Akita S., Nakamura R., Yamamoto N., Tokumoto H., Ishigaki T., Yamaji Y., Sasahara Y., Kubota Y., Mitsukawa N., Satoh K. (2016). Early Detection of Lymphatic Disorder and Treatment for Lymphedema following Breast Cancer. Plast. Reconstr. Surg..

[13] Ahmed (2006). Randomized controlled trial of weight training and lymphedema in breast cancer survivors (vol 24, pg 2765, 2006). J. Clin. Oncol..

[14] Meeske K.A., Sullivan-Halley J., Smith A.W., McTiernan A., Baumgartner K.B., Harlan L.C., Bernstein L. (2009). Risk factors for arm lymphedema following breast cancer diagnosis in Black women and White women. Breast Cancer Res. Treat..

[15] Shahpar H., Atieh A., Maryam A., Fatemeh H.S., Massoome N., Mandana E., Masud Y., Hamid Reza M., Mohammad Esmaeil A. (2013). Risk factors of lymph edema in breast cancer patients. Int. J. Breast Cancer.

[16] Gillespie T.C., Sayegh H.E., Brunelle C.L., Daniell K.M., Taghian A.G. (2018). Breast cancer-related lymphedema: Risk factors, precautionary measures, and treatments. Gland Surg..

[17] Das N., Baumgartner R., Riley E., Pinkston C., Yang D., Baumgartner K. (2015). Treatment-related risk factors for arm lymphedema among long-term breast cancer survivors. J. Cancer Surviv..

[18] Lopez Penha T.R., Van Roozendaal L.M., Smidt M.L., Boersma L.J., Von Meyenfeldt M.F., Voogd A.C., Heuts E.M. (2015). The changing role of axillary treatment in breast cancer: Who will remain at risk for developing arm morbidity in the future?. Breast.

[19] Tsai R.J., Dennis L.K., Lynch C.F., Snetselaar L.G., Zamba G.K.D., Scott-Conner C. (2009). The risk of developing arm lymphedema among breast cancer survivors: A meta-analysis of treatment factors. Ann. Surg. Oncol..

[20] Thomis S., Devoogdt N., Bechter-Hugl B., Nevelsteen I., Neven P., Fourneau I. (2020). Impact of a compression garment, on top of the usual care, in patients with breast cancer with early disturbance of the lymphatic transport: Protocol of a randomised controlled trial. BMJ Open.

[21] Devoogdt N., Lemkens H., Geraerts I., Van Nuland J., Flour M., Coremans T., Christiaens M.R., Van Kampen M. (2010). A new device to measure upper limb circumferences: Validity and reliability. Int. Angiol..

[22] Taylor R., Jayasinghe U.W., Koelmeyer L., Ung O., Boyages J. (2006). Reliability and validity of arm volume measurements for assessment of lymphedema. Phys. Ther..

[23] Damstra R.J., Glazenburg E.J., Hop W.C.J. (2006). Validation of the inverse water volumetry method: A new gold standard for arm volume measurements. Breast Cancer Res. Treat..

[24] Craig C.L., Marshall A.L., Sjöström M., Bauman A.E., Booth M.L., Ainsworth B.E., Pratt M., Ekelund U., Yngve A., Sallis J.F. (2003). International physical activity questionnaire: 12-country reliability and validity. Med. Sci. Sports Exerc..

[25] Thomis S., Devoogdt N., De Vrieze T., Bechter-Hugl B., Heroes A.K., Fourneau I. (2022). Relation Between Early Disturbance of lymphatic transport Visualized With Lymphofluoroscopy and Other Clinical Assessment Methods in Patients With Breast Cancer. Clin. Breast Cancer.

[26] Jørgensen M.G., Toyserkani N.M., Hansen F.C.G., Thomsen J.B., Sørensen J.A. (2021). Prospective Validation of Indocyanine Green Lymphangiography Staging of Breast Cancer-Related Lymphedema. Cancers.

[27] Akita S., Mitsukawa N., Rikihisa N., Kubota Y., Omori N., Mitsuhashi A., Tate S., Shozu M., Satoh K. (2013). Early diagnosis and risk factors for lymphedema following lymph node dissection for gynecologic cancer. Plast. Reconstr. Surg..

[28] Liu M., Liu S., Zhao Q., Cui Y., Chen J., Wang S. (2022). Using the Indocyanine Green (ICG) Lymphography to Screen Breast Cancer Patients at High Risk for Lymphedema. Diagnostics.

[29] Aldrich M.B., Rasmussen J.C., DeSnyder S.M., Woodward W.A., Chan W., Sevick-Muraca E.M., Mittendorf E.A., Smith B.D., Stauder M.C., Strom E.A. (2022). Prediction of breast cancer-related lymphedema by dermal backflow detected with near-infrared fluorescence lymphatic imaging. Breast Cancer Res. Treat..

[30] Mihara M., Hara H., Araki J., Kikuchi K., Narushima M., Yamamoto T., Iida T., Yoshimatsu H., Murai N., Mitsui K. (2012). Indocyanine Green (ICG) lymphography is superior to lymphoscintigraphy for diagnostic imaging of early lymphedema of the upper limbs. PLoS ONE.

[31] Akita S., Mitsukawa N., Kazama T., Kuriyama M., Kubota Y., Omori N., Koizumi T., Kosaka K., Uno T., Satoh K. (2013). Comparison of lymphoscintigraphy and indocyanine green lymphography for the diagnosis of extremity lymphoedema. J. Plast. Reconstr. Aesthetic Surg. JPRAS.

[32] Dean L.T., Kumar A., Kim T., Herling M., Brown J.C., Zhang Z., Evangelisti M., Hackley R., Kim J., Cheville A. (2016). Race or Resource? BMI, Race, and Other Social Factors as Risk Factors for Interlimb Differences among Overweight Breast Cancer Survivors with Lymphedema. J. Obes..

[33] Swaroop M.N., Ferguson C.M., Horick N.K., Skolny M.N., Miller C.L., Jammallo L.S., Brunelle C.L., O’Toole J.A., Isakoff S.J., Specht M.C. (2015). Impact of adjuvant taxane-based chemotherapy on the development of breast cancer-related lymphedema: Results from a large prospective cohort. Breast Cancer Res. Treat..

[34] Ugur S., Arici C., Yaprak M., Mesci A., Arici G.A., Dolay K., Ozmen V. (2013). Risk factors of breast cancer-related lymphedema. Lymphat. Res. Biol..

[35] Showalter S.L., Brown J.C., Cheville A.L., Fisher C.S., Sataloff D., Schmitz K.H. (2013). Lifestyle risk factors associated with arm swelling among women with breast cancer. Ann. Surg. Oncol..

[36] Ozaslan C., Kuru B. (2004). Lymphedema after treatment of breast cancer. Am. J. Surg..

[37] Haddad P., Farzin M., Amouzegar-Hashemi F., Kalaghchi B., Babazadeh S., Mirzaei H.R., Mousavizadeh A., Harirchi I., Rafat J. (2010). A multicentre cross-sectional study of arm lymphedema four or more years after breast cancer treatment in Iranian patients. Breast Cancer.

[38] Lane K.N., Dolan L.B., Worsley D., McKenzie D.C. (2007). Upper extremity lymphatic function at rest and during exercise in breast cancer survivors with and without lymphedema compared with healthy controls. J. Appl. Physiol..

[39] Baumann F.T., Reike A., Hallek M., Wiskemann J., Reimer V. (2018). Does Exercise Have a Preventive Effect on Secondary Lymphedema in Breast Cancer Patients Following Local Treatment?—A Systematic Review. Breast Care.

[40] Kwan M.L., Darbinian J., Schmitz K.H., Citron R., Partee P., Kutner S.E., Kushi L.H. (2010). Risk factors for lymphedema in a prospective breast cancer survivorship study: The Pathways Study. Arch. Surg..

[41] Paskett E.D., Herndon J.E., Day J.M., Stark N.N., Winer E.P., Grubbs S.S., Pavy M.D., Shapiro C.L., List M.A., Hensley M.L. (2008). Applying a conceptual model for examining health-related quality of life in long-term breast cancer survivors: CALGB study 79804. Psychooncology.

[42] Kilbreath S.L., Refshauge K.M., Ward L.C., Kastanias K., Yee J., Koelmeyer L.A., Beith J.M., French J.R., Ung O.A., Black D. (2013). Factors affecting the preoperative and postoperative extracellular fluid in the arm on the side of breast cancer: A cohort study. Lymphat. Res. Biol..

[43] Khanna S., Gupta A.K., Cherian A.J., Yadav B., Jacob P.M. (2019). Post Mastectomy Lymphedemaa Prospective Study of Incidence and Risk Factors. Indian J. Surg..

[44] Monleon S., Murta-Nascimento C., Bascuas I., Macià F., Duarte E., Belmonte R. (2015). Lymphedema predictor factors after breast cancer surgery: A survival analysis. Lymphat. Res. Biol..

[45] Ferguson C.M., Swaroop M.N., Horick N., Skolny M.N., Miller C.L., Jammallo L.S., Brunelle C., O’Toole J.A., Salama L., Specht M.C. (2016). Impact of ipsilateral blood draws, injections, blood pressure measurements, and air travel on the risk of lymphedema for patients treated for breast cancer. J. Clin. Oncol..

[46] Ben Salah H., Bahri M., Jbali B., Guermazi M., Frikha M., Daoud J. (2012). Upper limb lymphedema after breast cancer treatment. Cancer/Radiotherapie.

[47] De Luca A., Tripodi D., Frusone F., Leonardi B., Cerbelli B., Botticelli A., Vergine M., D’Andrea V., Pironi D., Sorrenti S. (2020). Retrospective Evaluation of the Effectiveness of a Synthetic Glue and a Fibrin-Based Sealant for the Prevention of Seroma Following Axillary Dissection in Breast Cancer Patients. Front. Oncol..

[48] Kwan W., Jackson J., Weir L.M., Dingee C., McGregor G., Olivotto I.A. (2002). Chronic arm morbidity after curative breast cancer treatment: Prevalence and impact on quality of life. J. Clin. Oncol..

[49] Ashikaga T., Krag D.N., Land S.R., Julian T.B., Anderson S.J., Brown A.M., Skelly J.M., Harlow S.P., Weaver D.L., Mamounas E.P. (2010). Morbidity results from the NSABP B-32 trial comparing sentinel lymph node dissection versus axillary dissection. J. Surg. Oncol..

[50] Donker M., van Tienhoven G., Straver M.E., Meijnen P., van de Velde C.J.H., Mansel R.E., Cataliotti L., Westenberg A.H., Klinkenbijl J.H.G., Orzalesi L. (2014). Radiotherapy or surgery of the axilla after a positive sentinel node in breast cancer (EORTC 10981-22023 AMAROS): A randomised, multicentre, open-label, phase 3 non-inferiority trial. Lancet Oncol..

[51] Fontaine C., Adriaenssens N., Vanparijs H., Voordeckers M., Jean-François F., De Coster L., Schallier D., Vanhoeij M., Verfaillie G., Lamote J. (2011). A prospective analysis of the incidence of breast cancer related lymphedema of the arm after surgery and axillary lymph node dissection in early breast cancer patients treated with concomitant irradiation and anthracyclines followed by paclitaxel. Eur. J. Lymphology Relat. Probl..

[52] Cariati M., Bains S., Grootendorst M., Suyoi A., Peters M., Mortimer P., Ellis P., Harries M., Van Hemelrijck M., Purushotham A. (2015). Adjuvant taxanes play a key role in the development of upper limb breast cancer-related lymphoedema. Eur. J. Surg. Oncol..

[53] Aoishi Y., Oura S., Nishiguchi H., Hirai Y., Miyasaka M., Kawaji M., Shima A., Nishimura Y. (2020). Risk factors for breast cancer-related lymphedema: Correlation with docetaxel administration. Breast Cancer.

[54] Gross J.P., Sachdev S., Helenowski I.B., Lipps D., Hayes J.P., Donnelly E.D., Strauss J.B. (2018). Radiation Therapy Field Design and Lymphedema Risk After Regional Nodal Irradiation for Breast Cancer. Int. J. Radiat. Oncol. Biol. Phys..

[55] Kibar S., Dalyan Aras M., Ünsal Delialioğlu S., Köseoğlu B.F. (2015). A cross-sectional study examining the risk factors associated with lymphedema and its prevalence in breast cancer patients after level 3 axillary lymph node dissection. Turkiye Fiziksel Tip ve Rehabilitasyon Dergisi.

